# Effect of repeated intratracheal instillation of incense smoke condensate in mice

**DOI:** 10.1371/journal.pone.0331098

**Published:** 2025-09-02

**Authors:** In-Hyeon Kim, Je-Hein Kim, Se-Woong Park, Han Na Suh, Su-Jin Lim, Min-Sung Kang, Hyeon-Young Kim, Dong Im Kim, Moonjung Hyun, Sung-Hwan Kim

**Affiliations:** 1 Division of Jeonbuk Advanced Bio Research, Korea Institute of Toxicology, Jeongeup, Jeollabuk-do, Republic of Korea; 2 College of Veterinary Medicine, Chonnam National University, Gwangju, Republic of Korea; 3 Korea Occupational Safety and Health Agency, Daejeon, Republic of Korea; 4 Division of Gyeongnam Bio-Environmental Research, Bio-Health Research Center, Gyeongsangnam-do, Republic of Korea; Rutgers Biomedical and Health Sciences, UNITED STATES OF AMERICA

## Abstract

Incense smoke condensate (ISC) can have harmful mutagenic and genotoxic effects. Epidemiological and experimental studies have reported the negative effects of incense use on humans. We investigated the toxicological effects of the incense smoke condensate ISC in a 2-week repeated intratracheal instillation model in mice. Twenty-five male mice were divided into four treatment groups and one control group (*n* = 5 per group). The treatment groups received daily intratracheal instillations of ISC at doses of 2.5, 5, 10, and 20 mg/kg/day, and the control group received a vehicle control for the duration of the study. Mortality and body weight were recorded during the study period. At the end of the study, all mice were sacrificed and terminal body weight, organ weight, gross findings, total and differential cell counts in the bronchoalveolar lavage fluid (BALF), and histopathological findings were obtained. Lung inflammatory markers were measured using quantitative real-time polymerase chain reaction. The results showed that ISC exposure led to dose-dependent increases in both absolute and relative left lung weights, as well as in the number of total cells, macrophages, and neutrophils in BALF. Furthermore, the ISC significantly elevated the mRNA expression levels of inflammatory markers such as IL-1β, IL-6, TNF-α, and MMP-12 in the lung tissues in a dose-dependent manner. Histopathological analysis revealed significant changes in the lungs, including epithelial hyperplasia, inflammatory cell infiltration, and macrophage aggregation. These findings indicate that ISC induces lung inflammation. The no-observed-adverse-effect level of ISC was determined to be less than 2.5 mg/kg/day in this mouse model.

## 1. Introduction

Incense comprises a combustible mixture of wood, seeds, resins, spices, roots, and synthetic chemicals [1–4]. It is widely used in various forms, including sticks, coils, powder, cones, and ropes, to produce fragrances that enhance mood and ambiance [3,5]. Incense burning is a traditional practice in many Southeast Asian and Middle Eastern countries, where it is frequently used during religious rituals or to create a fragrant indoor environment [1–4]. Incense use is prevalent among Southeast Asians with approximately 50% of the population regularly burning incense for worship [4]. Moreover, over 90% of households in the United Arab Emirates use incense at least once a week [6].

Despite its widespread use, the potential health risks associated with incense smoke exposure have emerged. Associations between frequent incense burning and adverse health effects, including an increased risk of leukemia and respiratory diseases have been reported [7–10]. Ho et al. [7] reported that temple workers exposed to incense smoke exhibited a higher prevalence of chronic and acute irritative symptoms such as persistent cough and nasal or throat irritation. Animal studies have demonstrated that incense smoke exposure can induce morphological changes in the lung tissue, neutrophil infiltration, and increased pro-inflammatory cytokine expression, including TNF-α and IL-4 [11–13]. Additionally, incense smoke condensate (ISC) exhibits genotoxic effects, such as sister chromatid exchange, with toxicity levels exceeding those of tobacco smoke in mammalian cells [14].

However, conflicting findings have been reported regarding the association between incense smoke exposure and health risks. Some studies, such as those conducted by Chan-Yeung et al. [15] and Koo et al. [16], found no significant correlation between incense smoke exposure and lung cancer or chronic respiratory disease. This discrepancy highlights the need for further studies to elucidate the toxicological effects of incense smoke in controlled conditions. Although previous studies have primarily focused on long-term exposure and in vitro models, standardized in vivo studies evaluating the effects of short-term exposure remain limited. Additionally, dose–response studies investigating ISC toxicity in well-established animal models are lacking.

To address these gaps, the present study aimed to evaluate the potential toxicological effects of ISC using a controlled in vivo model. Mice were exposed to daily intratracheal instillation of ISC for 2 weeks under Good Laboratory Practice conditions. Unlike previous studies that predominantly relied on long-term observational data, this study systematically assessed the effects of short-term ISC exposure by integrating lung tissue analysis, bronchoalveolar lavage fluid (BALF) cellular composition, histopathological changes, and cytokine expression. By conducting this study under standardized laboratory conditions, we aimed to elucidate the mechanisms underlying ISC-induced lung toxicity, which could serve as a foundation for future mechanistic studies. Furthermore, this study sought to quantify the no-observed-adverse-effect level (NOAEL) of ISC exposure, thus providing valuable data for risk assessment.

## 2. Materials and methods

### 2.1. Preparation of ISC

**Fig 1:**
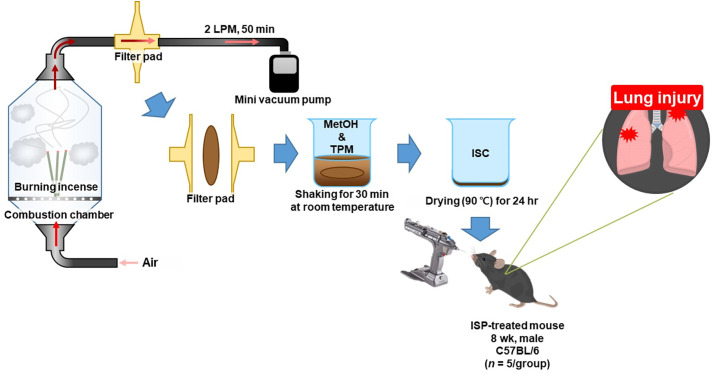
Schematic diagram of simulation combustion chamber with sampling attachments. TPM, trapped particle matter.

This study used a commonly available incense stick from a South Korean brand. Each incense stick was 240 mm long and 4 mm in circumference. As shown in Fig 1, the ISC samples were prepared by burning the incense in a combustion chamber (dimensions: cubes 30 cm × 30 cm × 30 cm; pyramids 30 cm × 30 cm × 13.2 cm). Incense smoke was collected on a Whatman Cambridge Filter Pad (Whatman Grade F319-04 filter paper; GE Healthcare, Buckinghamshire, UK) at 2 LPM for 50 min using a mini-vacuum pump (XR5000; SKC Inc, Eighty Four, USA). To obtain the ISC, the trapped particulate matter on the filter pad was extracted by shaking with absolute methanol for 30 min at room temperature. After extraction, the solvent was evaporated under reduced pressure in a dry oven at 90 °C for 24 h. The extracted and dried incense matter was used as the ISC sample for subsequent animal studies.

### 2.2. ISC analysis

The ISC components were analyzed using a gas chromatograph (GC-2010; Shimadzu, Kyoto, Japan) equipped with a mass spectrometer (GCMS-QP2010 ultra; Shimadzu, Kyoto, Japan). For the analysis, the ISC was dissolved in distilled water containing 5% dimethyl sulfoxide (DMSO) and 1.0 µL of this solution was introduced into the gas chromatography injector at 280 °C using the auto-injector (AOC-5000; Shimadzu, Kyoto, Japan). The target analytes were then transferred using a carrier gas (He > 99.999%, flow rate of 1.0 mL/min, and constant flow) to a DB-5MS separation column (diameter: 0.25 mm, length: 30 m, and thickness: 0.25 µm, Agilent, Santa Clara, USA). The gas chromatography oven temperature was initially set to 40 °C (for 5 min) and then ramped at 5 °C/min to 300 °C, where it was maintained for 3 min, resulting in a total run time of 60 min. The interface and ion source temperatures were set to 280 °C to enhance the detection of ISC components. The components were examined using the total ion chromatogram mode over a mass range of 35–500 m/z. Detailed information on the instrument system is provided in Table 1.

**Table 1:** Operational settings of the GC-MS system during the ISC sample analysis.

GC (GC-2010; Shimadzu, Kyoto, Japan) and MS (GCMS-QP2010 ultra; Shimadzu, Kyoto, Japan)			
Column: DB-5MS (diameter: 0.25 mm, length: 30 m, and film thickness: 0.25 μm)			
Oven setting:		Detector setting	
Oven temperature	40 °C (5 min)	Ionization mode	EI (70 eV)
Oven rate	5 °C/min	Ion source temperature	280 °C
Maximum oven temperature	300 °C (3 min)	Interface temperature	280 °C
Total time	60 min	TIC scan range	35–500 m/z
Auto injector (AOC-5000; Shimadzu, Kyoto, Japan)			
Injector temperature	280 °C	Purge flow	3.0 mL/min
Carrier gas	Helium (> 99.999%)	Split ratio	5.0
Column flow	1.0 mL/min		

GC, gas chromatography; MS, mass spectrometry; EI, electron ionization; TIC, total ion chromatography.

### 2.3. Animal husbandry and maintenance

Seven-week-old male C57BL/6 mice were purchased from Orient Bio Inc. (Seongnam, Republic of Korea) and used after a one-week quarantine and acclimatization period. Each animal was housed individually in a cage within a room maintained at a temperature of 23 ± 3 °C, relative humidity of 50 ± 20%, and light intensity of 150–300 lux, with a 12-hour light/dark cycle; ventilation was used to refresh the air 10–20 times/h. The mice were fed pelleted food (Lab Diet^®^ #5053; PMI Nutrition International, Richmond, USA) and UV-irradiated (Steritron SX-1; Daeyoung Inc., Seoul, Republic of Korea) and filtered (pore size, 1 μm) tap water was provided *ad libitum*. All experimental procedures were approved by the Institutional Animal Care and Use Committee of the Korea Institute of Toxicology (IACUC #1901−0023).

### 2.4. Experimental groups and treatment dose

**Fig 2:**
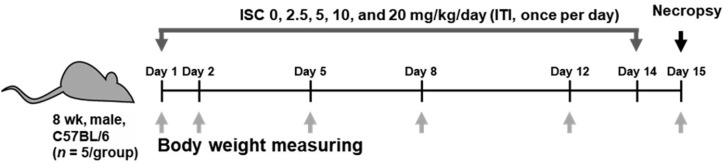
Animal experimentation schedule. ITI, intratracheal instillation.

A total of 25 healthy male mice were randomly assigned to five experimental groups: four treatment groups (**n* *= 5/group) receiving 2.5, 5, 10, and 20 mg/kg/day ISC, and a vehicle control (VC) group receiving 5% DMSO in phosphate-buffered saline (PBS) through intratracheal instillation. In this study, doses of 2.5, 5, 10, and 20 mg/kg/day of ISC were selected to investigate potential adverse effects, using a common ratio of 2 between dose levels. The ISC was dissolved in 5% DMSO in PBS and prepared daily prior to treatment. The dosing solution concentration was calculated based on the most recently recorded body weight of an individual animal. The ISC was administered daily through intratracheal instillation to the mice for 2 weeks at a dose volume of 50 μL using an automatic video instillator. The VC group received an equivalent volume of 5% DMSO in PBS through the same route (Fig 2).

### 2.5. Clinical observation, mortality, and body weight

All animals were monitored for mortality and clinical signs twice daily (before and after treatment) throughout the study period. Additionally, body weight was measured on days 1, 2, 5, 8, and 12 after treatment commenced, and the terminal body weight was recorded on day 15 during necropsy (Fig 2). Changes in the general appearance, abnormal behavior, and weight loss, were considered signs of toxicity.

### 2.6. Gross findings

All animals were anesthetized with isoflurane (Ifran Liquid; Hana Pharm Co., Ltd, Hwaseong, Republic of Korea) on day 15. The mice were sacrificed by exsanguination of the abdominal aorta. Gross examination of the lungs was performed on all sacrificed animals.

### 2.7. BALF and cell differentiation

The right lung was prepared for BALF collection by cannulating the trachea using 0.7 mL PBS three times in each mouse. The total number of cells in BALF was counted using a NucleoCounter (NC-250; ChemoMetec, Gydevang, Denmark). The BALF was centrifuged (Shandon Cytospin 4; Thermo Scientific, Waltham, USA) at 800 rpm for 10 min and the supernatant was placed on a glass slide. Differential cell counts were determined by counting 200 cells/slide, including macrophages, neutrophils, lymphocytes, and eosinophils. The cells were stained with Diff-Quik solution (Sysmex, Kobe, Japan) and observed under a light microscope (Leica DM 2500; Leica Microsystems, Wetzlar, Germany) at 400 × magnification. After BALF analysis, the right lung of each animal was stored in a deep freezer (below −90 °C) until quantitative real time-PCR (qRT-PCR) analysis

### 2.8. Organ weight

On day 15, the left lung (which had not undergone BALF collection), heart, liver, spleen, and kidneys were weighed, and the relative weight was calculated (organ-to-terminal body weight ratios).

### 2.9. qRT-PCR

Total RNA was isolated from right lung tissues using an RNeasy Mini Kit (Qiagen, Valencia, USA), according to the manufacturer’s protocol, and quantified using a NanoDrop 2000 spectrophotometer (Thermo Scientific, Wilmington, USA). Reverse transcription was performed using 500 ng of total RNA and the First Strand cDNA Synthesis Kit (Takara, Kyoto, Japan) according to the manufacturer’s instructions. Mouse gene-specific primer sequences used are listed below: glyceraldehyde 3-phosphate dehydrogenase (GAPDH; forward, 5′-ATCACCATCTTCCAGGAGCGA-3′; reverse, 5′-AGGGGCCATCCACAGTCTT-3′), interleukin-1 beta (IL-1β; forward, 5′-GGGCCTCAAAGGAAAGAATC-3′; reverse, 5′-TACCAGTTGGGGAACTCTGC-3′), interleukin-6 (IL-6; forward, 5′-AGACTTCCATCCAGTTGCCT-3′; reverse, 5′-CAGGTCTGTTGGGAGTGGTA-3′), tumor necrosis factor alpha (TNF-α; forward, 5′-ACGGCATGGATCTCAAAGAC-3′; reverse, 5′-GTGGGTGAGGAGCACGTAGT-3′), and matrix metalloproteinase-12 (MMP-12; forward, 5′-CACAACAGTGGGAGAGAAAA-3′; reverse, 5′-AGCTTGAATACCAGATGGGATG-3′). qRT-PCR was performed using Power SYBR^®^ Green Master Mix (Applied Biosystems, Foster City, USA) with the StepOnePlus™ Real-Time PCR Systems (Applied Biosystems). The expression level of each transcript was normalized to the internal control gene (GAPDH). Relative gene expression was calculated using the ^ΔΔ^Ct method, where Ct = threshold cycle.

### 2.10. Histopathological findings

After weighing the organs were fixed in 10% neutral-buffered formalin for 24 h. The tissues were routinely processed, embedded in paraffin, and sectioned at a thickness of 4 µm. These sections were stained using hematoxylin and eosin (Sigma-Aldrich, St. Louis, MO, USA) for histopathological examination. The sections were examined under a light microscope at 200 × magnification. Histopathological changes, including epithelial hyperplasia, mononuclear cell and neutrophil infiltration, and macrophage aggregation, were scored on a scale from 0 to 5. The scoring was performed as follows: 0, no symptoms; 1, minimal (< 20%); 2, slight (20–40%); 3, moderate (40–60%); 4, Marked (60–80%); 5, Severe (> 80%). Each successive field was assessed individually to determine the incidence and severity of tissue damage.

### 2.11. Statistics

Statistical analyses were performed using the GraphPad Prism 10 software (GraphPad Software Inc., San Diego, CA, USA). Results are expressed as mean ± standard deviation (SD). Comparisons among groups were performed using one-way analysis of variance (ANOVA), followed by Dunnett’s post hoc test to compare with the VC group, with significance levels denoted by *p < 0.05, **p < 0.01, and ***p < 0.001. Additionally, Tukey’s post hoc test was applied for multiple comparisons, with *p* < 0.05 considered statistically significant.

## 3. Results

### 3.1. ISC analysis

GC-MS analysis was performed on the ISC samples (Table 2). This analysis detected 30 organic compounds in the ISC, which accounted for 99.96% of the extract after adjusting for background components from the solvent. Among these, three abundant primary components were identified. Levoglucosan, a compound produced from the combustion of cellulose, comprised the largest proportion (30.46%) of ISC extract. Acetophenone, a compound with a pleasant scent that is often found in fragrances, accounted for 8.05%. The third major component was 2,6-dimethoxyphenol, which represented 7.24% of the extract. These findings highlight the chemical complexity of ISC and suggest that the high presence of certain compounds, such as levoglucosan, may play a key role in the biological effects associated with incense smoke.

**Table 2:** List of the major organic compounds detected in the ISC sample.

Retention time (min)	Compound name	Molecular weight (g/mol)	Formula	Peak area (%)
13.43	Phenol	94	C6H6O	0.87
13.66	2-Hydroxy-gamma-butyrolactone	102	C4H6O3	0.66
16.84	2-Methoxyphenol	124	C7H8O2	0.61
17.36	3-Hydroxypyridine	95	C5H5NO	2.38
17.02	Pentanal	86	C5H10O	2.95
18.93	Dihydro-4-hydroxy-2(3H)-furanone	102	C4H6O3	1.62
19.87	5-Hexyldihydro-2(3H)-furanone	170	C10H18O2	2.38
20.18	Catechol	110	C6H6O2	4.01
20.70	1,4;3,6-Dianhydro-alpha-d-glucopyranose	144	C6H8O4	3.44
20.87	2,3-Dihydrobenzofuran	120	C8H8O	0.67
21.10	2,3-Anhydro-d-mannosan	144	C6H8O4	0.90
22.14	Sucrose	342	C12H22O11	5.01
24.52	2,6-Dimethoxyphenol	154	C8H10O3	7.24
25.81	Vanillin	152	C8H8O3	1.52
26.28	4-Hydroxy-2-butanone	88	C4H8O2	0.79
27.03	1,2,3-Trimethoxybenzene	168	C9H12O3	2.61
27.03	1,2,4-Trimethoxybenzene	168	C9H12O3	2.61
27.21	3-Allyl-6-methoxyphenol	164	C10H12O2	0.50
28.06	Acetovanillone	166	C9H10O3	2.90
28.47	Levoglucosan	162	C6H10O5	30.46
28.98	5-tert-Butylpyrogallol	182	C10H14O3	0.97
29.09	1-(4-Hydroxy-3-methoxyphenyl)-2-propanone	180	C10H12O3	0.70
29.47	Tetraacetate	363	C13H17NO11	2.19
29.98	Acetophenone	180	C10H12O3	8.05
30.66	Diethyl phthalate	222	C12H14O4	3.40
30.83	2,6-Dimethoxy-4-(2-propenyl)-phenol	194	C11H14O3	1.59
31.94	Homovanillic acid	182	C9H10O4	2.59
33.78	Acetosyringone	196	C10H12O4	3.67
33.98	4-((1E)-3-Hydroxy-1-propenyl)-2-methoxyphenol	180	C10H12O3	1.67
38.14	5,10-Diethoxy-2,3,7,8-tetrahydro-1H,6H-dipyrrolo[1,2-a:1’,2’-d] pyrazine	250	C14H22N2O2	1.00

Organic components in the collected ISC samples. Solvent blank correction (excluding organic components detected in 5% DMSO).

### 3.2. Clinical observations, mortality rates, body weights, and gross findings

Clinical observations, mortality rates, and body weight were assessed to monitor the general health effects of ISC exposure. No treatment-related signs of distress, adverse clinical symptoms, or mortality were observed in any of the ISC-treated groups, suggesting that ISC exposure did not cause any noticeable acute toxicity or health issues in animals. Additionally, body weight measurements showed no significant differences between the VC and ISC-treated groups, indicating that ISC exposure did not affect the overall growth or weight maintenance (S1 Fig). However, during the scheduled necropsy, visual examination of the lung tissue revealed notable changes across all ISC-treated groups. Specifically, red or pale discoloration was observed on the surface of the lungs of animals exposed to ISC, regardless of the dosage (S1 Table). These discolorations suggest potential localized effects on the lung tissue that may indicate inflammatory or other pathological responses. Together, these findings imply that although ISC exposure did not lead to overt clinical toxicity or weight changes, lung tissue alterations were observed in all treated groups.

### 3.3. Total and differential cell counts in the BALF

**Fig 3:**
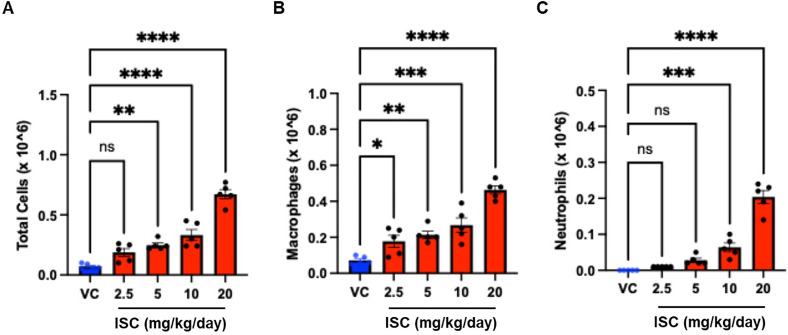
Effect of ISC on the total and differential cell counts in the BALF. The number of total cells (A), macrophages (B), and neutrophils (C) in the BALF of the VC and ISC-treated groups. Data are presented as the mean ± SD (*n* = 5/group). ^*^p < 0.05, ^**^p < 0.01, ^***^p < 0.001, compared with the VC group; one-way ANOVA with Tukey’s post hoc test.

The total and differential cell counts in the BALF are shown in Fig 3; the total number of cells in the BALF was significantly increased in the 5, 10, and 20 mg/kg/day ISC-treated groups compared to that in the VC group (Fig 3A). The number of macrophages in the BALF was significantly increased in all ISC-treated groups compared to that in the VC group (Fig 3B). The number of neutrophils in BALF was significantly higher in the 10 and 20 mg/kg/day groups than in the VC group (Fig 3C). No significant differences in the number of lymphocytes and eosinophils were observed between the VC- and ISC-treated groups (S1 Fig).

### 3.4. Organ weight

**Fig 4:**
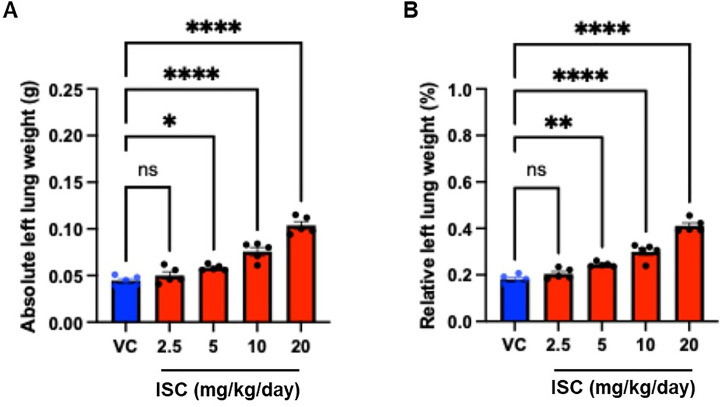
Effect of the ISC on absolute and relative left lung weight in mice. Data are presented as the mean ± SD (*n* = 5/group). ^*^p < 0.05, ^**^p < 0.01, ^***^p < 0.001; compared with the VC group one-way ANOVA with Tukey’s post hoc test.

Fig 4 presents the results for both the absolute and relative weights of the left lung in response to ISC treatment. The data showed that left lung weights, both in absolute (total weight) and relative terms (weight as a percentage of body weight), were significantly higher in the groups treated with ISC at doses of 5, 10, and 20 mg/kg/day than in the VC group. This increase in lung weight suggests a possible inflammatory or pathological response in the lung tissue, potentially due to ISC exposure. In contrast, the weights of other major organs, including the heart, liver, spleen, and kidneys, did not show significant differences between the ISC-treated groups and the VC group (S2 Fig) This observation suggests that ISC exposure may specifically impact the lung tissue without causing notable changes in the weight of other organs.

### 3.5. mRNA expression levels of inflammatory genes

**Fig 5:**
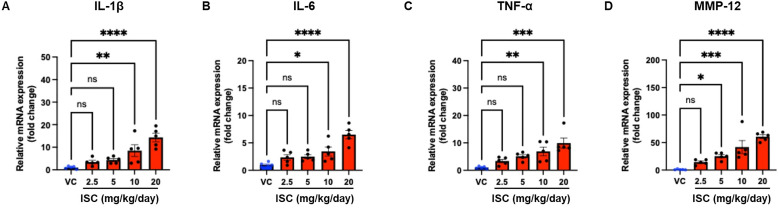
Effect of the ISC on the mRNA levels of inflammatory genes in the right lung tissues. The expression levels of IL-1β (A), IL-6 (B), TNF-α (C), and MMP-12 (D) were determined using qRT-PCR. The values were normalized to GAPDH and expressed as fold changes. Data are presented as the mean ± SD (*n* = 5/group). ^*^p < 0.05, ^**^p < 0.01, ^***^p < 0.001, compared with the VC group; one-way ANOVA with Tukey’s post hoc test.

Analysis of the mRNA expression levels of inflammatory genes in the right lung tissues, as presented in Fig 5, indicated that exposure to ISC led to a significant increase in the expression of key inflammatory markers. Specifically, the expression mRNA levels of IL-1β, IL-6, and TNF-α were notably elevated in the groups treated with ISC at doses of 10 and 20 mg/kg/day compared to the VC group (Figs 5A-C). This result suggests that exposure to higher ISC concentrations triggers an inflammatory response because these genes are critical mediators of inflammation. Additionally, the expression levels of MMP-12, an enzyme associated with tissue remodeling and inflammatory lung diseases, were significantly higher in all ISC-treated groups (5, 10, and 20 mg/kg/day) than in the VC group (Fig 5D). This consistent increase across all doses indicates that even low levels of ISC exposure may influence lung tissue remodeling and inflammatory processes. These findings collectively suggest that ISC exposure can activate inflammatory pathways in the lung, with a dose-dependent increase in the expression of certain inflammatory genes.

### 3.6. Histopathological findings

**Fig 6:**
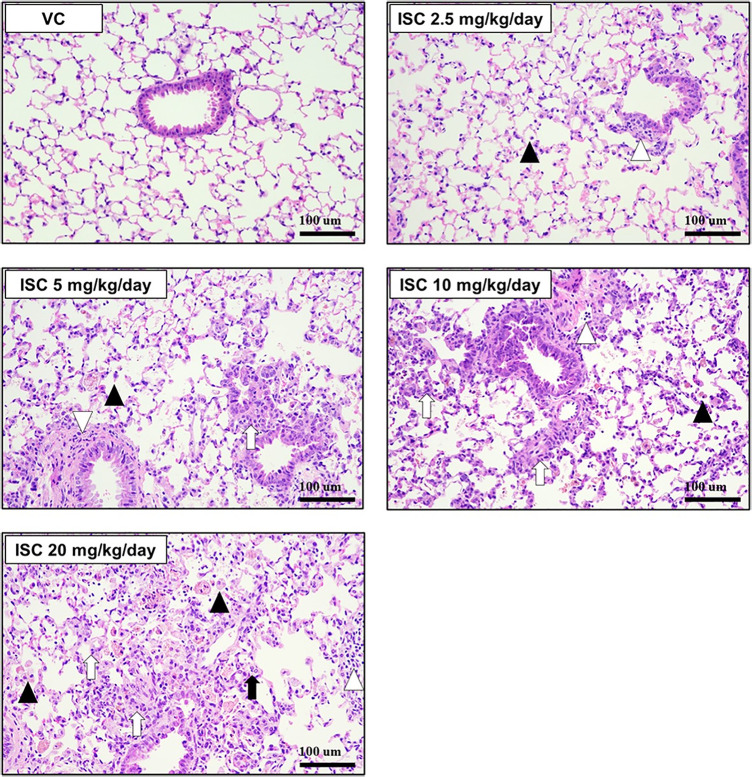
Effect of the ISC on histopathological findings of the left lung tissues. VC group lung section showing normal appearance and the ISC-treated groups showing various histopathological alterations characterized by epithelial hyperplasia (white arrows), mononuclear cell infiltration to peribronchiolar/perivascular (white arrowheads), neutrophil infiltration to alveolar (black arrows), and pigmented/foamy macrophage aggregates (black arrowheads). Scale bar indicates 100 μm (original magnification, 200 ×).

The histopathological analysis results, presented in Fig 6 and Table 3, provide insight into the structural changes in the lung tissue following ISC exposure. In the VC group, the lungs maintained a normal architecture with no signs of abnormal tissue changes. A scoring system ranging from 0 to 5 was used to evaluate the degree of lung injury (Table 3). This scoring helped quantify the severity of the various observed changes. In the ISC-treated groups, several specific lung alterations were noted compared to those in the VC group. Overall, these histopathological findings demonstrate that ISC exposure results in notable inflammatory and structural changes in lung tissue, including epithelial cell proliferation, immune cell infiltration, and macrophage aggregation. These effects were more pronounced at higher ISC doses, indicating a dose-dependent relationship with lung injury severity.

**Table 3:** Quantitative histopathological findings of the lung tissues.

Parameters	Grades	VC	ISC (mg/kg/day)			
			2.5	5	10	20
Epithelial hyperplasia	Normal	5	4	0	0	0
	Minimal	0	1	3	1	0
	Slight	0	0	2	4	2
	Moderate	0	0	0	0	3
	Mean ± SD	0.0 ± 0.00	0.2 ± 0.45	1.4 ± 0.55^**^	1.8 ± 0.45^**^	2.6 ± 0.55^**^
Mononuclear cell infiltration to peribronchiolar/ perivascular	Normal	5	0	0	0	0
	Minimal	0	5	1	0	0
	Slight	0	0	4	5	3
	Moderate	0	0	0	0	2
	Mean ± SD	0.0 ± 0.00	1.0 ± 0.00^**^	1.8 ± 0.45^**^	2.0 ± 0.00^**^	2.4 ± 0.55^**^
Neutrophil infiltration to alveolar	Normal	5	5	5	2	0
	Minimal	0	0	0	3	4
	Slight	0	0	0	0	1
	Mean ± SD	0.0 ± 0.00	0.0 ± 0.00	0.0 ± 0.00	0.6 ± 0.55^*^	1.2 ± 0.45^**^
Pigmented/foamy macrophage aggregates	Normal	5	0	0	0	0
	Minimal	0	1	0	0	0
	Slight	0	4	0	0	0
	Moderate	0	0	5	4	1
	Marked	0	0	0	1	4
	Mean ± SD	0.0 ± 0.00	1.8 ± 0.45^**^	3.0 ± 0.00^**^	3.2 ± 0.45^**^	3.8 ± 0.45^**^

0, no symptoms; 1, minimal; 2, slight; 3, moderate; 4, marked; 5, severe.

Data are presented as the mean ± SD (**n* *= 5/group). * *p* < 0.05, ** *p* < 0.01, compared with the VC group.

## 4. Discussion

### 4.1. ISC exposure induces lung inflammation and weight increase

The present study investigated the potential toxicological effects of ISC from a 2-week repeated intratracheal instillation in C57BL/6 mice at doses of 0, 2.5, 5, 10, and 20 mg/kg/day. The results of this study showed that ISC increased lung inflammatory events, including increased weight of lung, increased inflammatory cell and cytokine level in the BALF, and morphological changes owing to the inflammatory response in the lung.

Typically, changes in organ weight are sensitive indicators of toxic chemicals [17–20]. Several studies have demonstrated that incense smoke exposure was correlated with increased lung weight [21,22]. These results were consistent with the data from our study, which showed that a 2-week repeated intratracheal instillation of ISC led to a significant increase in lung weight. Hussain et al. [23] reported that long-term (4, 8 or 13-week) exposure to incense smoke induced toxicity in kidney function and architecture, as well as in the lungs. In this study, no treatment-related changes were observed weight in the heart, liver, spleen, or kidney weights. Interestingly, after 2 weeks of exposure, changes in weight were observed only in the lungs. Therefore, our findings suggest that the lung are a major target organ of ISC toxicity after short-term exposure. Although our study was limited to a single experiment, the application of multiple concentrations of ISC enabled a more comprehensive, dose-dependent evaluation of the toxicological effect of incense smoke exposure. This approach provides valuable insights into the relationship between exposure levels and lung injury, thereby enhancing our understanding of the health risks associated with exposure to incense smoke.

### 4.2. ISC exposure triggers inflammatory cell infiltration in the BALF

Incense smoke inhalation induces lung injury and increases the number of inflammatory cells in BALF [24]. These results are consistent with the data from our study showing that the number of total cells, macrophages, and neutrophils in BALF increased after ISC instillation in a dose-dependent manner. Alveolar inflammation is induced by air pollutants, as ultrafine particles, urban PM2.5 pollutants, and microscopic airborne particulates of asbestos and silica from building materials, and that these pollutants also exacerbate lung disease [25–27]. Upon reaching a specific threshold or overload, the ultrafine particles deposited in the alveolar region of the lungs can overwhelm the antioxidant defense of air-way cells, leading to the onset of oxidative stress and an accompanying inflammatory reaction [28–32]. Our results suggest that the increased number of inflammatory cells in the BALF was caused by the inhalation of particulate matter into the lungs.

### 4.3. Histopathological changes in lung tissue following ISC exposure

Our histopathological analysis revealed that ISC exposure induced significant alterations in lung tissue, including neutrophil, macrophage, and mononuclear cell infiltration. Similar findings have been reported in other studies, where incense smoke exposure led to epithelial hyperplasia and peribronchiolar mononuclear cell infiltration in mice as well as neutrophil and pigmented macrophage accumulation in the alveolar region of rats [21,33,34]. Alarifi et al. [33] reported that exposure to incense for 14 weeks induced pneumocyte hyperplasia in rats, which caused thickening of the alveolar walls via deposition of collagen fibrils in the alveolar walls. Concurrent to previous findings, our results demonstrated that continuous exposure to incense smoke does not degrade the lungs, but may lead to collagen deposition owing to the increased inflammatory responses in the lungs and damage to lung epithelial cell.

### 4.4. Increased expression of pro-inflammatory cytokines in the lung

Pro-inflammatory cytokines such as IL-1β, IL-6, and TNF-α play crucial roles in the early stages of lung inflammation, while MMP-12 is crucial in lung inflammatory development [35–40]. Consistent with our findings, Niu et al. [41] reported that incense exposure upregulates pro-inflammatory cytokines (e.g., TNF-α, IL-6) in A549 cells. In our study, ISC instillation led to increased expression of inflammatory cytokines in lung tissues, further confirming their pro-inflammatory effects. Cytokine expression was assessed using PCR, a widely accepted method for evaluating gene expression changes in inflammation-related pathways. Although ELISA can provide direct quantification of secreted cytokines in the BALF, PCR analysis offers insight into transcriptional regulation and upstream inflammatory signaling. Given that inflammatory responses are regulated at both transcriptional and protein levels, our approach effectively identified ISC-induced inflammatory responses.

### 4.5. Toxic organic compounds in ISC and their respiratory Effects

In our study, 30 organic compounds, including levoglucosan, acetophenone, 2,6-dimethoxyphenol, aldehydes, and phthalates, were identified in the ISC. Diethyl phthalate is a respiratory toxicant that promotes inflammatory cytokine secretion and contributes to asthma and allergic diseases upon inhalation [42,43]. Similarly, aldehydes aggravate the respiratory system and exacerbate conditions such as asthma and chronic obstructive pulmonary disease [44]. Benzene, a volatile organic compound, is associated with nasopharyngeal cancer and structural lung changes [3]. Even at low concentrations, inhalation of these toxic compounds can cause significant damage to alveolar epithelial cells. Our results suggest that the exposure to these compounds from the ISC may directly contribute to lung inflammation and respiratory toxicity.

## 5. Conclusion and implications for human health

In conclusion, our results indicate that a 2-week repeated intratracheal instillation of ISC induces significant lung inflammation, as evidenced by increased lung weight, inflammatory cell infiltration, elevated levels of inflammatory cytokines, and histopathological changes. These findings suggest that ISC toxicity primarily targets the lungs under short-term exposure. The presence of particulate matter, organic compounds, and gaseous byproducts in ISC likely contributes to these inflammatory effects. Under the conditions of this study, the NOAEL for ISC in male mice was < 2.5 mg/kg/day, considering the observed mononuclear cell infiltration and increased macrophage aggregation at this dose. These findings provide valuable insight into the potential health risks associated with incense smoke exposure in humans.

The ISC utilized in this study was collected via a filtration-based method, which inherently limited the capture of the volatile and gaseous components present in the incense smoke. Therefore, the toxicological effects observed in this study may not fully reflect those associated with direct inhalation of incense smoke in real-world settings. Additionally, incense formulations vary across regions and cultures, which may influence toxicological outcomes. Future studies should consider comparative analyses using incense from different geographical regions to comprehensively assess the potential variations in toxicity and associated health risks. Furthermore, pathological evaluation of other organs would be beneficial in determining whether incense exposure results in systemic toxicity beyond the lungs.

## Supporting information

S1 Fig Body weight and Lymphocyte and eosinophil in the BALF. (A) Effect of the ISC on body weight in mice. Data are presented as the mean ± SD (*n* = 5/group). Effect of the ISC on the total and differential cell counts in the BALF. The number of lymphocyte (B), and eosinophil (C) in the BALF of the VC group and ISC-treated groups. Data are presented as the mean ± SD (*n* = 5/group).
(PDF)

S2 Fig Absolute and relative heart, liver, spleen, and kidneys weight. Effect of the ISC on absolute and relative organs weight in mice. Data are presented as the mean ± SD (n = 5/group).
(PDF)

S1 Table Gross findings of the lung. (PDF)

S1 Data B219002 Body weight, Organ weight, Gross finding. (XLSX)

S2 Data B219002 Histopathology. (XLSX)

S3 Data B219002 Total & differential cell count. (XLSX)

S4 Data B219002_qPCR_GAPDH, IL-1b, IL-6, TNF-a, MMP-12. (XLS)
