# Supplementary material for: Effect of repeated intratracheal instillation of incense smoke condensate in mice
Source: PLoS One. 2025 Sep 2;20(9):e0331098. doi: 10.1371/journal.pone.0331098 (PMC12404431; doi:10.1371/journal.pone.0331098)
Supplement: S1 Fig — (A) Effect of the ISC on body weight in mice. Data are presented as the mean ± SD (n = 5/group). Effect of the ISC on the total and differential cell counts in the BALF. The number of lymphocyte (B), and eosinophil (C) in the BALF of the VC group and ISC-treated groups. Data are presented as the mean ± SD (n = 5/group). (PDF) [file pone.0331098.s001.pdf]

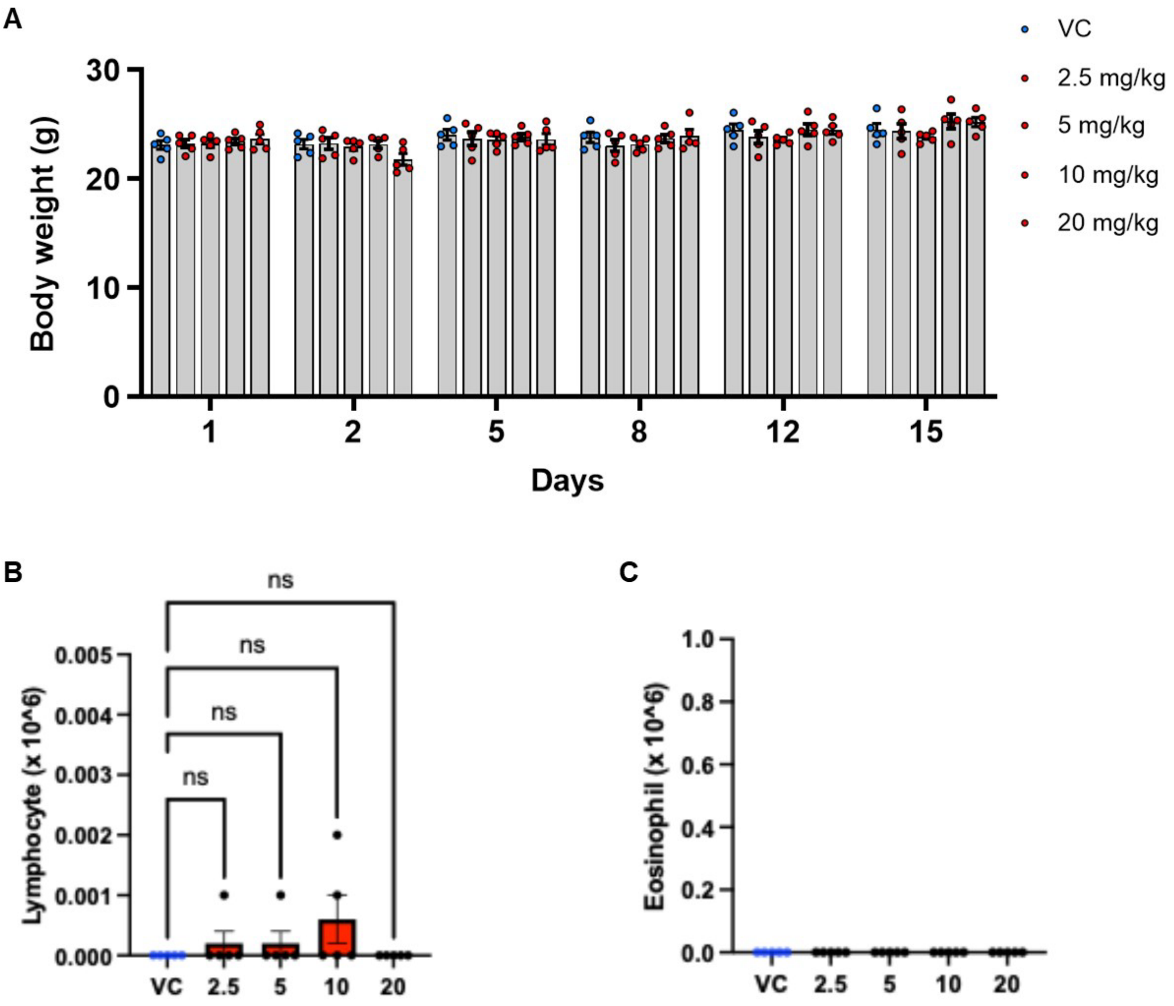

S1 Fig. Body weight and Lymphocyte and eosinophil in the BALF

(A) Effect of the ISC on body weight in mice. Data are presented as the mean ± SD (*n* = 5/group). Effect of the ISC on the total and differential cell counts in the BALF. The number of lymphocyte (B), and eosinophil (C) in the BALF of the VC group and ISC-treated groups. Data are presented as the mean ± SD (*n* = 5/group).
