# Supplementary material for: Effect of repeated intratracheal instillation of incense smoke condensate in mice
Source: PLoS One. 2025 Sep 2;20(9):e0331098. doi: 10.1371/journal.pone.0331098 (PMC12404431; doi:10.1371/journal.pone.0331098)
Supplement: S2 Fig — Effect of the ISC on absolute and relative organs weight in mice. Data are presented as the mean ± SD (n = 5/group). (PDF) [file pone.0331098.s002.pdf]

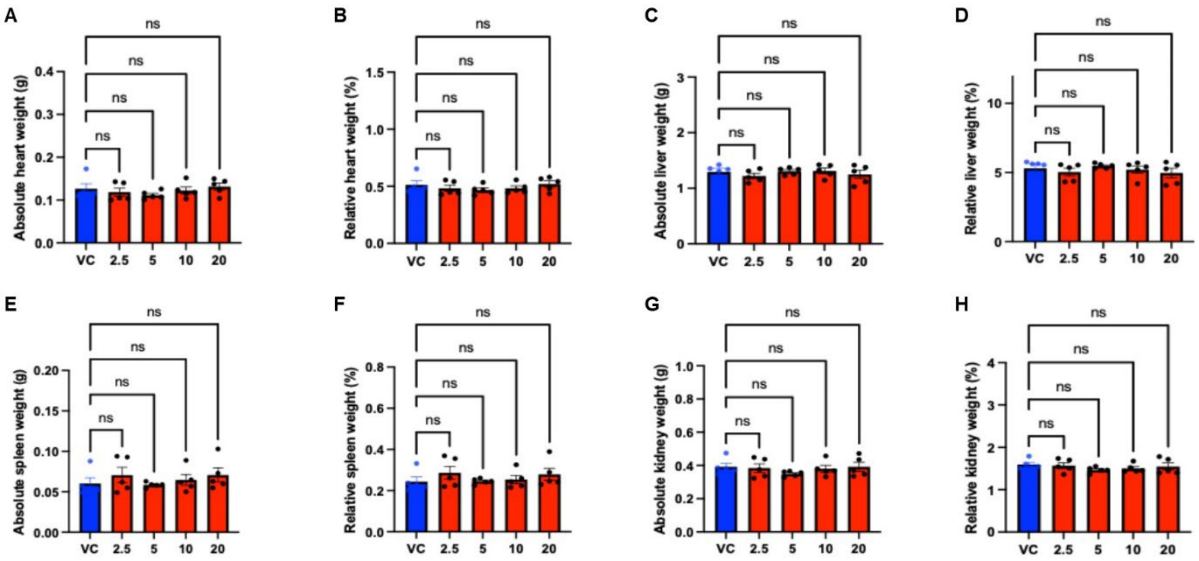

S2 Fig. Absolute and relative heart, liver, spleen, and kidneys weight

Effect of the ISC on absolute and relative organs weight in mice. Data are presented as the mean ± SD (*n* = 5/group).
