# Supplementary material for: Effect of repeated intratracheal instillation of incense smoke condensate in mice
Source: PLoS One. 2025 Sep 2;20(9):e0331098. doi: 10.1371/journal.pone.0331098 (PMC12404431; doi:10.1371/journal.pone.0331098)
Supplement: S1 Table — (PDF) [file pone.0331098.s003.pdf]

S1 Table. Gross findings of the lung

Category		VC	ISC (mg/kg/day)			
			2.5	5	10	20
No. of mice		5	5	5	5	5
Lung	Normal	5	0	0	0	0
	All lobe partial red	0	5	5	4	2
	All lobe partial pale	0	0	0	1	2
	Left lobe partial pale/ right lobe partial red	0	0	0	0	1
